# Shoulder disorders in an outpatient clinic: an epidemiological study

**DOI:** 10.1590/1413-785220172503170849

**Published:** 2017

**Authors:** Eduardo Angeli Malavolta, Mauro Emilio Conforto Gracitelli, Jorge Henrique Assunção, Gustavo de Mello Ribeiro Pinto, Arthur Zorzi Freire da Silveira, Arnaldo Amado Ferreira

**Affiliations:** Universidade de São Paulo, Faculdade de Medicina, Hospital das Clínicas, Instituto de Ortopedia e Traumatologia, Shoulder and Elbow Group, São Paulo, SP, Brazil

**Keywords:** Shoulder, Prevalence, Diagnosis, Rotator cuff

## Abstract

**OBJECTIVE:**

To describe shoulder disorders in patients evaluated by two shoulder and elbow surgeons.

**METHODS:**

This cross-sectional study analyzed patients evaluated by two authors, excluding acute fractures and dislocations and patients with symptoms not involving the shoulder. Age and sex distribution was determined for the different diagnoses.

**RESULTS:**

We evaluated 1001 patients. Mean age was 51.43±15.15 years and 51.0% were female. Disorders of the rotator cuff occurred in 64.3% (41.2% tendinopathy, 11.0% partial tears and 12.2% full-thickness tears). Adhesive capsulitis occurred in 13.5% of cases and glenohumeral instability in 8.1%. Rotator cuff disorders were more common in women, with a peak between 50 and 59 years for tendinopathy and partial tears and between 60 and 69 years for full-thickness tears. Glenohumeral instability was more frequent in men, with a peak between 30 and 39 years.

**CONCLUSION:**

The most frequent diagnosis was rotator cuff tendinopathy, followed by adhesive capsulitis, full-thickness rotator cuff tears, partial rotator cuff tears and glenohumeral instability. Rotator cuff lesions were more common in women, with a peak between 60 and 69 years for full-thickness tears. ***Level of Evidence IV, Case Series.***

## RESUMO

**OBJETIVO:**

Descrever as afecções do ombro atendidas por dois membros da Sociedade Brasileira de Ombro e Cotovelo.

**MÉTODOS:**

Estudo transversal que avaliou atendimentos de dois dos autores. Excluímos fraturas e luxações agudas e pacientes com sintomatologia que não envolvia o ombro. A distribuição etária e por sexo foi determinada para os diferentes diagnósticos.

**RESULTADOS:**

Avaliamos 1001 pacientes. A idade foi de 51,43 ± 15,15 anos e 51,0% eram do sexo feminino. As afecções do manguito rotador ocorreram em 64,3%, sendo 41,2% de tendinopatia, 11,0% de rotura parcial e 12,2% de rotura completa. A capsulite adesiva ocorreu em 13,5% e instabilidade glenoumeral em 8,1%. As afecções do manguito rotador foram mais frequentes em mulheres, com pico entre 50 e 59 anos para tendinopatia e rotura parcial, e entre 60 e 69 anos para rotura completa. A instabilidade glenoumeral foi mais frequente em homens, com pico entre 30 e 39 anos.

**CONCLUSÃO:**

Os diagnósticos mais frequentes foram tendinopatia do manguito rotador, seguido de capsulite adesiva, rotura completa do manguito rotador, rotura parcial do manguito rotador e instabilidade glenoumeral. As afecções do manguito rotador foram mais frequentes em mulheres, com pico entre 60 e 69 anos para rotura completa. ***Nível de Evidência IV, Série de Casos.***

## INTRODUCTION

Shoulder complaints are frequent in the population, with an annual incidence of 14.7/1000^1^ and prevalence of 7 to 14%^2^ ^-^ ^4^ which rises to 67% in older people.^5^ Few studies have epidemiologically assessed patients with shoulder pain and described the main diagnostics.^1^ ^,^ ^6^ ^-^ ^10^

The most frequent causes of shoulder pain are tendinopathy of the rotator cuff and adhesive capsulitis.^8^ ^-^ ^10^ To our knowledge, no studies have examined the epidemiology of the main shoulder complaints in Brazil. Establishing the national panorama is useful for stimulating public education policies, alerting doctors about the problems that most affect the population and helping define plans for prevention and treatment. 

The objective of this study is to describe the various shoulder disorders treated in outpatients by two Brazilian shoulder and elbow surgeons, as well as to present the distribution of the main diagnoses by sex and age.

## METHODS

This cross-sectional study was conducted using data from patients treated by the two main authors (EAM and MECG). These researchers have 10 and 9 years of experience, respectively, in shoulder and elbow surgery. This study was approved by the institutional review board under process number 1195.

All individuals attended between July 1, 2015 and May 25, 2016 were included. Patients with acute fractures and dislocations were excluded, as well as those with symptoms which did not involve the shoulder. MRI or X-rays in conjunction with ultrasound were taken in all patients.

### Assessment methods

The database was constructed using FileMaker (FileMaker Incorporated, Santa Clara, CA, USA). This tool was used to create a spreadsheet in Excel (Microsoft Corporation, Redmond, WA, USA) containing data for age, sex and diagnosis. Age was recorded in years completed at the time of the first treatment and categorized into 10-year intervals. Diagnosis was classified as: tendinopathy of the rotator cuff, partial tear of the rotator cuff, complete tear of the rotator cuff, adhesive capsulitis, calcific tendonitis, glenohumeral instability, SLAP lesion (superior labrum anterior to posterior), glenohumeral osteoarthritis, acromioclavicular osteoarthritis, scapular dyskinesia, chronic acromioclavicular dislocation and other. The "other" category included those diagnoses which occurred in less than 0.5% of the sample. For cases with more than one diagnosis, the patient's electronic record was reviewed and only the most clinically significant diagnosis was considered. Distribution by age and by sex was determined for the most frequent diagnoses.

### Statistical analysis

The data are presented in a descriptive manner with absolute numbers and percentages. The general characteristics of the sample for age, sex and comorbidities were presented as means and standard deviation for continuous data and total amount and percentages for categorical data. Calculation was performed using SPSS 21.0 software (Chicago, IL, USA).

## RESULTS

We evaluated the medical records of 1338 patients. Of these, we excluded 64 with shoulder fractures, 33 with elbow fractures, 15 with acute acromioclavicular or sternoclavicular dislocation, 159 with orthopedic disorders of the elbow and 66 for orthopedic disorders in other sites, leaving 1001 patients with shoulder disorders.

Mean patient age for the sample was 51.43±15.15 years and 511 patients (51.0%) were female. The youngest patient was 10 years old and the oldest was 98.

Rotator cuff disorders accounted for 64.3% of cases, tendinopathy accounted for 41.2%, 11.0% were partial tears and 12.2% were complete tears. Adhesive capsulitis occurred in 13.5% of cases and glenohumeral instability in 8.1%. The distribution of shoulder disorders is shown in Table 1.

**Table 1:** Absolute and percentage distribution of diagnoses affecting the shoulder.

Diagnosis	n	%
Rotator cuff	644	64.3
Rotator cuff tendinopathy	412	41.2
Partial rotator cuff tear	110	11
Complete rotator cuff tear	122	12.2
Adhesive capsulitis	135	13.5
Shoulder instability	81	8.1
Calcific tendonitis	36	3.6
SLAP lesion	32	3.2
Glenohumeral arthrosis	22	2.2
Acromioclavicular joint arthrosis	14	1.4
Scapular dyskinesia	14	1.4
Chronic acromioclavicular dislocation	8	0.8
Others	15	1.5
Total	1001	100

SLAP: superior labrum anterior to posterior.

**Figure 1:**
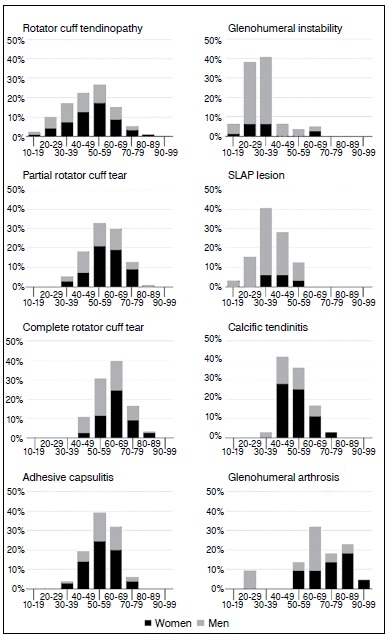
Percentage distribution of main diagnoses by decade of life and sex.

Rotator cuff disorders were more frequent in women, with tendinopathy and partial tears peaking between 50 and 59 years and complete tears peaking between 60 and 69 years. Adhesive capsulitis and calcific tendonitis were also more frequent in female patients, with the former peaking between 50 and 59 years and the latter between 40 and 49 years. Shoulder instability and SLAP lesion prevailed in young men, peaking between 30 and 39 years. Glenohumeral arthritis occurred mainly after 50 years of age and involved more females. Distribution of cases by the various age groups and by sex for each of the main diagnoses is presented in Figure 1.

## DISCUSSION

Our results show that rotator cuff disorders were present in 64.1% of the sample, with 41.2% tendinopathy, 11.0% partial tears and 12.2% complete tears. Rotator cuff tendinopathy, the most frequent diagnosis in our sample, was also the most prevalent diagnosis in studies by Juel and Natvig^8^ and Ostör et al.,^(10 )^while Walker-Bone et al.^9^ reported that the most frequent disorder was adhesive capsulitis. Tendinopathy and partial tear peaked at 50-59 years; tendinopathy presented a wider distribution, while partial tear was only observed after 30 years of age. Complete rotator cuff tear was most prevalent between 60 and 69 years and occurred only after 40 years of age. These findings are consistent with the progressive nature of rotator cuff injuries and the fact that complete tears increase in prevalence with age.^11^ Rotator cuff disorders were more common in women, except for tendinopathy in the 20 to 39 year age group, partial tears in the 40 to 49 year group and complete tears in the 40 to 59 year group. Juel and Natvig^8^ also found greater involvement in women, but more men had complete tears between ages 50 and 59. We believe that this may be at least partly explained by a greater number of men performing manual labor. Our data, however, do not allow us to draw this conclusion.

Adhesive capsulitis was present in 13.5% of patients, the second most frequent diagnosis. As with the findings by Juel and Natvig,^8^ this diagnosis predominated in women and peaked between 50 and 59 years of age. It did not occur under age 30 or above age 80. Similar frequency has been reported by other authors, with a variation of 11 to 16%.^8^ ^,^ ^10^ The fifth most frequent diagnosis was glenohumeral instability, occurring in 8.1% of the sample. As Juel and Natvig also found,^8^ this diagnosis was predominant in young men.

Our study showed that calcific tendonitis involved 3.6% of the sample and was prevalent in women 40 to 59 years old. The other studies did not individualize this diagnosis and probably included patients with this diagnosis in within rotator cuff tendinopathy or other disorders.^1^ ^,^ ^8^ ^-^ ^10^ SLAP lesions caused symptoms in 3.2% of our series and also were not separated by the other authors.^1^ ^,^ ^8^ ^-^ ^10^ Juel and Natvig^8^ studied labral lesions and glenohumeral instability together, while the other studies did not describe this diagnosis.^1^ ^,^ ^9^ ^,^ ^10^ We noted that this disease and glenohumeral instability were more prevalent in young men. Glenohumeral osteoarthritis was seen in 2.2% of the patients we studied, 4% lower than the number described by Juel and Natvig.^8^ The other studies do not describe this condition in detail.^1^ ^,^ ^9^ ^,^ ^10^ With the exception of 2 patients, our data demonstrate that this condition predominantly affects women after age 50, peaking between 60 and 69 years. Juel and Natvig^8^ found all cases of this condition after age 40 years and a peak after age 70. We emphasize that glenohumeral osteoarthritis represents 37.5% (6/16) of the diagnoses in patients aged 80 or older. The two young patients with glenohumeral osteoarthritis in our series had a specific diagnosis of arthropathy secondary to juvenile rheumatoid arthritis and septic arthritis.

We chose to present the main diagnosis in cases with more than one disease. The purpose of this methodology was to facilitate data analysis and understanding. A similar methodology has been used by other authors^8^ ^,^ ^9^ and may affect some results, especially for acromioclavicular osteoarthritis, joint degeneration that which affects up to 82% of asymptomatic individuals.^12^ However, we chose to highlight clinical findings over changes in imaging. Unlike Juel and Natvig^(8 )^but like other authors,^9^ ^,^ ^10^ we chose not to consider myalgia a diagnosis in itself. We believe that myalgia is more of a symptom than a specific diagnosis^(8 )^and that it may present concurrently with other disorders.

This study has limitations. We did not include cases of acute fractures and dislocations; we opted to exclude these patients because these injuries are generally treated in the emergency unit. Consequently, only less serious cases come to the outpatient clinic, along with those that do not remain hospitalized for surgical treatment. While this sample may not be statistically representative of the entire national population, it is larger than the majority of similar studies.^1^ ^,^ ^8^ ^,^ ^10^ Additionally, the patients were seen in a private practice by specialists, so this data may not be generalized to patients in the Brazilian Unified Health System and to general orthopedists, decreasing external validity. However, we emphasize that all patients were personally assessed by one of the main authors (EAM and MECG), who are surgeons with ample experience diagnosing these disorders and imagery was confirmed via MRI or a combination of x-ray and ultrasound. These characteristics increase the internal validity of the data.

## CONCLUSIONS

The most common diagnoses in the specialist clinic were tendinopathy of the rotator cuff (41.2%), adhesive capsulitis (13.5%), complete rotator cuff tear (12.2%), partial rotator cuff tear (11.0%) and glenohumeral instability (8.1%). Rotator cuff disorders were more frequent in women, with tendinopathy and partial tears peaking between 50 and 59 years and complete tears peaking between 60 and 69 years. Adhesive capsulitis was more frequent in female patients, peaking between 50 and 59 years old, while glenohumeral instability was more frequent in men and peaked between 30 and 39 years of age.
